# Reaction diffusion system prediction based on convolutional neural network

**DOI:** 10.1038/s41598-020-60853-2

**Published:** 2020-03-03

**Authors:** Angran Li, Ruijia Chen, Amir Barati Farimani, Yongjie Jessica Zhang

**Affiliations:** 1 Carnegie Mellon University, Department of Mechanical Engineering, Pittsburgh, 15213 United States; 2 Carnegie Mellon University, Department of Electrical and Computer Engineering, Pittsburgh, 15213 United States

**Keywords:** Mechanical engineering, Computational science

## Abstract

The reaction-diffusion system is naturally used in chemistry to represent substances reacting and diffusing over the spatial domain. Its solution illustrates the underlying process of a chemical reaction and displays diverse spatial patterns of the substances. Numerical methods like finite element method (FEM) are widely used to derive the approximate solution for the reaction-diffusion system. However, these methods require long computation time and huge computation resources when the system becomes complex. In this paper, we study the physics of a two-dimensional one-component reaction-diffusion system by using machine learning. An encoder-decoder based convolutional neural network (CNN) is designed and trained to directly predict the concentration distribution, bypassing the expensive FEM calculation process. Different simulation parameters, boundary conditions, geometry configurations and time are considered as the input features of the proposed learning model. In particular, the trained CNN model manages to learn the time-dependent behaviour of the reaction-diffusion system through the input time feature. Thus, the model is capable of providing concentration prediction at certain time directly with high test accuracy (mean relative error <3.04%) and 300 times faster than the traditional FEM. Our CNN-based learning model provides a rapid and accurate tool for predicting the concentration distribution of the reaction-diffusion system.

## Introduction

Reaction-diffusion systems have attracted a considerable amount of attention in recent years. They arise naturally in various chemistry models to describe the spatiotemporal concentration change of one or more chemical species which involve both local chemical reaction and diffusion simultaneously. Chemical reactions transform substances from one to another and the diffusion process causes substances to spread out over the spatial domain. The reaction-diffusion system consists of a set of partial differential equations (PDEs) to represent the behaviour of each chemical species individually. The solution of reaction-diffusion systems also presents many interesting phenomena such as spatial patterns, moving fronts or pulses and oscillations^1–4^. Therefore, it has been used to study complex chemical or biological processes including multiple chemical components such as combustion theory^5,6^, calcium dynamics^7^ and nerve impulse propagation^8^. The diversity of spatial patterns in reaction-diffusion systems also inspires the biological pattern formation study^9–11^ and spatial ecological study^12,13^.

**Figure 1:**
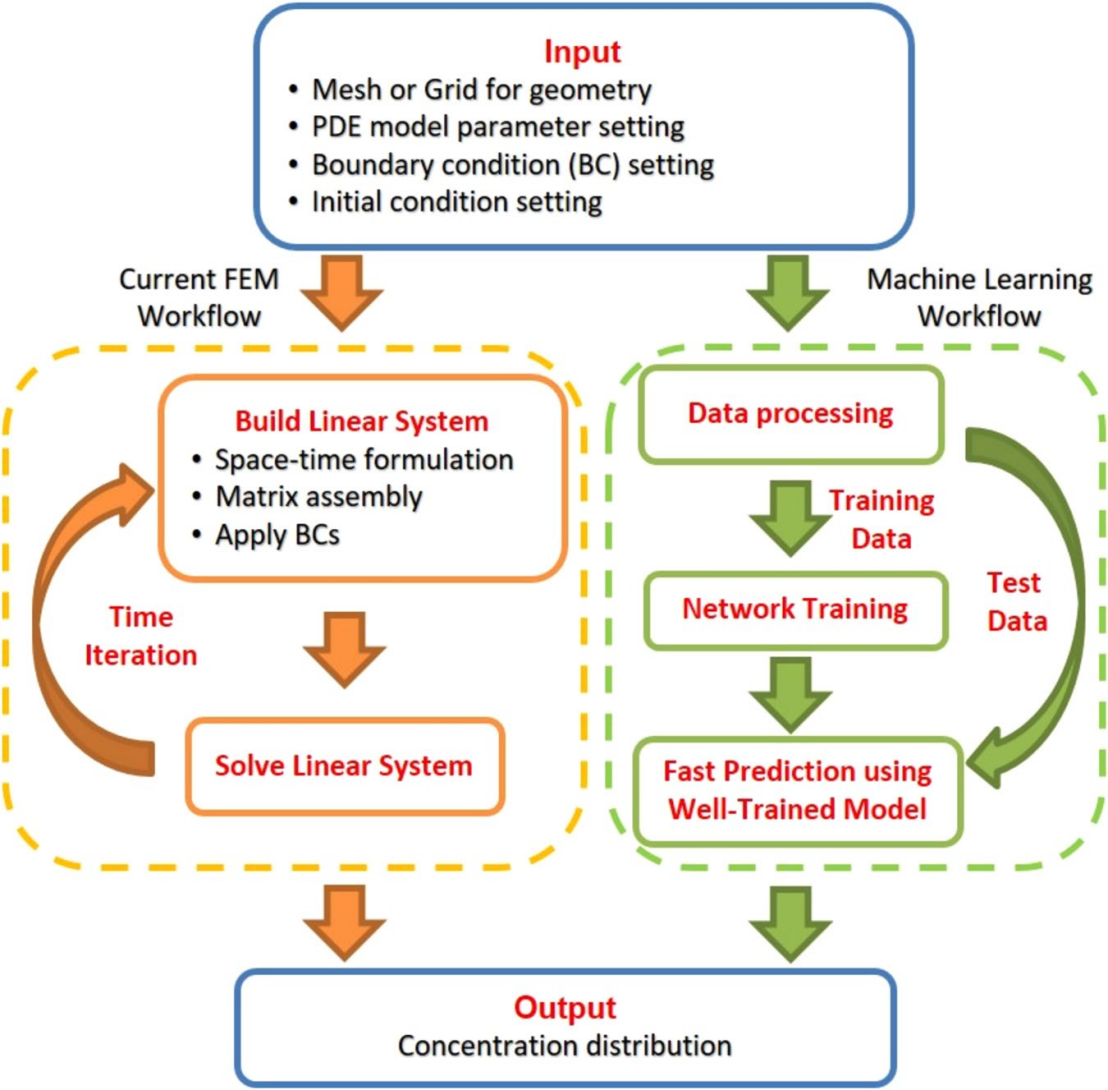
The current FEM workflow versus a machine learning-based solution for concentration prediction of a reaction-diffusion system.

Numerical methods such as Finite Difference Method (FDM) and Finite Element Method (FEM) are widely used to derive the approximate solution of the reaction-diffusion system^14–16^. The current FEM workflow for solving a time-dependent reaction-diffusion system is shown in Fig. 1: (i) generating a mesh corresponding to the geometry; (ii) setting up the finite element model by specifying diffusion and reaction coefficients and the initial and boundary conditions; and (iii) iteratively assembling and solving the linear equations until the expected computational error is reached. The complexity and time-consuming nature of the current FEM workflow makes it hard to rapidly generate output feedback to the input setting. This limits its application to simulate a reaction-diffusion system which occurs in a large spatial domain and requires long time to reach the steady state. Without rapid feedback, it is also inconvenient to synthesize the desired spatial pattern which needs multiple input adjustments of the system.

To address limitations in the current FEM workflow, we use deep learning to study spatial distribution of the reaction-diffusion system and provide quick and accurate prediction given different parameters (Fig. 1). In recent years, deep learning has been proven successful in areas of speech recognition^17,18^ and computer vision^19,20^. In particular, different convolutional neural networks (CNN) are designed and lead to extensive application in image recognition and segmentation^21,22^ as well as video classification^23^. The use of encoder-decoder architecture in designing neural networks is a major trend now to establish straightforward mapping between input features and output results. The architecture has been widely used in CNN and achieves great performance in machine translation^24–26^, image semantic segmentation^27^ and road scene understanding^28^. The practical success of deep learning in artificial intelligence also inspires new algorithm in solving high-dimensional PDEs^29^ and discovering PDEs following the laws of physics^30^. In addition, the combination of machine learning and FEM is also attractive to improve the performance of the conventional FEM workflow^31,32^ and develop data-driven solvers^33^. A deep learning model was designed to directly estimate stress distributions of the aorta^34^. The conditional generative adversarial network (*cGAN*) was also applied in a data-driven paradigm for rapid inference, modeling and simulation of transport phenomena^35^.

In this paper, we propose to predict the spatial distribution result of a specific reaction-diffusion system using an encoder-decoder based CNN. The reaction-diffusion system is based on Zeldovich equation controlled by several simulation parameters. In our CNN model, the encoder is defined using a four-layer CNN that takes the input of different simulation parameters, boundary conditions, geometry configurations, and output a reduced feature vector. The decoder is another four-layer CNN that takes the feature vector from the encoder and predicts the concentration distribution. The well-trained learning model can predict concentration distributions within 1 second with an average error of 3.04% compared to the traditional FEM. By introducing time as the input feature, our model learns the time-dependent behaviour of the reaction-diffusion system and predicts dynamic concentration distributions within a certain time range. Our model also manages to give accurate prediction even when the distribution is complex or changes rapidly due to complex geometry and simulation settings. Therefore, our CNN-based learning model provides a rapid and accurate tool for predicting the concentration distribution of the reaction-diffusion system.

## Results

### FEM simulation of the reaction-diffusion system

**Figure 2:**
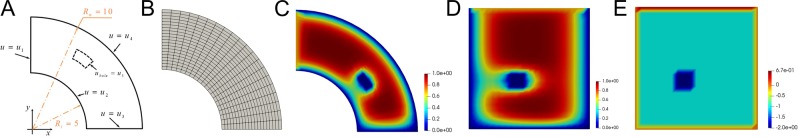
The reaction-diffusion system in our model. (**A**) The problem setting of the reaction-diffusion system. Black dash box represents a hole with a fixed size. (**B**) The quadrilateral mesh used in FEM simulations. (**C**) The concentration result in the physical domain. (**D**) The concentration result in the parametric domain. (**E**) The boundary condition visualized in the parametric domain.

In this paper, we study a one-component reaction-diffusion system inside a one-fourth arc pipe in 2D domain (Fig. 2A). To consider the effect of different geometry configurations, we introduce a hole with fixed size at different locations of the pipe (black dash box in Fig. 2A). We perform FEM simulations to obtain the spatiotemporal distribution results (Fig. 2C) of different geometries and parameter settings. The FEM results are then processed to become the input dataset of our model (Fig. 2D,E). See the section of Method for more details about the problem setting and data generation.

### Network architecture

**Figure 3:**
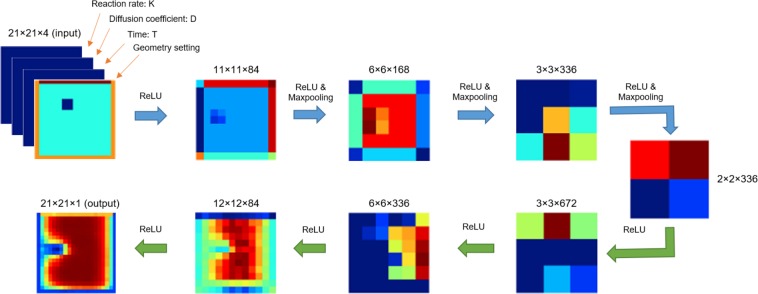
The convolutional neural network with encoder-decoder architecture. Blue and green arrows represent encoding and decoding, respectively. A representative output for each layer is shown for both encoding and decoding layers.

In our model, we design and implement a CNN since it can extract features from our tensor-format input data. We refer to the convolutional encoder-decoder architecture to design the network as shown in Fig. 3. The input four-channel tensor depicting geometry, boundary conditions and parameter settings are processed by a series of convolutional operations in the encoder network to downsample the tensor and obtain a low-dimensional representation. Then, the decoder network reverses the operations in the encoder by a series of deconvolutional operations to upsample the reduced representation and generate the predicted solution. In Fig. 3, the top row (encoder network) shows how the optimized features are extracted through a series of convolutional layers. For instance, in the 11 × 11 feature map, a region looking like the hole is distinguishable from the other interior region and the four boundary edges show different colors from the interior region indicating different boundary values. After one convolutional operation, the resulting 6 × 6 feature map becomes abstract but the hole feature is still noticeable. Therefore, it is believed that optimal information is continuously extracted layer by layer so that the geometry and parameter settings are transformed to the distribution results properly. The bottom row (decoder network) in Fig. 3 shows how the extracted features are reconstructed in the corresponding distribution results.

To select the appropriate activation function in the network, we test networks for three widely used activation functions: *ReLU*^36^, *PReLU*^37^ and *ELU*^38^. The test results illustrate that all three activation functions can achieve similar accuracy while ReLU needs the shortest time in training (see Supplemental Information; Fig. S1). Therefore, we employ ReLU as the activation function in both the encoder and the decoder.

Moreover, *maxpooling* is used in the encoder process to drop unnecessary features to prevent overfitting and keep transition and rotation invariance of features. During the learning process, we use the Mean Square Error (MSE) as the loss function to adjust the weights of all layers,

1 $$MSE=\frac{1}{M\times N}\mathop{\sum }\limits_{j=1}^{M}\mathop{\sum }\limits_{i=1}^{N}{({u}_{i,j}^{P}-{u}_{i,j}^{G})}^{2},$$

where ${u}_{i,j}^{P}$ is the *i*^*th*^ entity of the *j*^*th*^ predicted concentration matrix, while ${u}_{i,j}^{G}$ is the *i*^*th*^ entity of the *j*^*th*^ ground truth concentration matrix. In our training process, we set the learning rate to be 0.0001. We also monitor the loss function value in Equation (1) for each training epoch to make sure the training converges.

### Model evaluation

We use the mean absolute error (MAE) and the mean relative error (MRE) as two performance metrics to evaluate the accuracy of the predicted concentration distributions. For each predicted result, the MAE is defined by

2 $$MAE=\sqrt{\frac{1}{N}\mathop{\sum }\limits_{i=1}^{N}{({u}_{i}^{P}-{u}_{i}^{G})}^{2}},$$

where *N* denotes the number of entities in the output matrix, ${u}_{i}^{P}$ and ${u}_{i}^{G}$ denote the *i*^*th*^ entity in the prediction matrix and the ground truth matrix, respectively. For each predicted result, the MRE is defined by

3 $$MRE=\frac{MAE}{\max \left|\left|{u}^{G}\right|\right|-\min \left|\left|{u}^{G}\right|\right|}\times 100 \% ,$$

where $\max \left|\left|{u}^{G}\right|\right|$ and $\min \left|\left|{u}^{G}\right|\right|$ denote the maximum and minimum values from the ground truth matrix, respectively.

**Figure 4:**
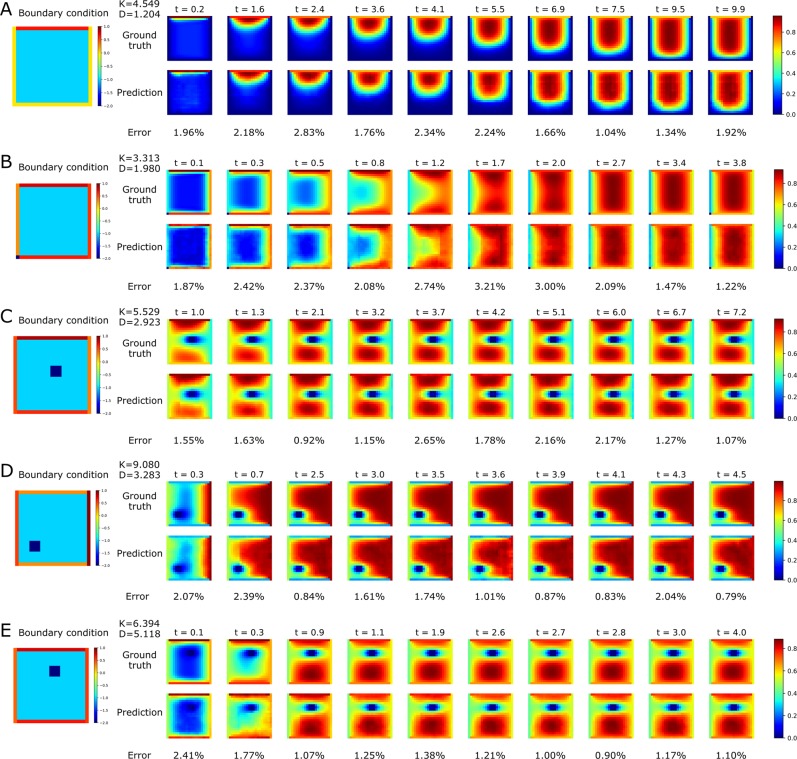
The concentration distribution comparison with five different simulation configurations. (**A**,**B**) The same geometry without hole; and (**C**–**E**) geometries with hole at three different locations. The examples are selected in test dataset to show the prediction performance for different distribution profiles. For each configuration, the ground truth results, predicted results and errors are shown from top to bottom.

We first study the effect of the original state and main parameters *D*, *K* and time *t* on the dynamic concentration pattern of the reaction-diffusion system. The dataset consists of 2,500 matrices, each of which was 21 by 21. After establishing the architecture of our neural network, we train the network by randomly selecting 75% samples as the training data. Our models are trained for 100 epochs and the MSE loss converges to 0.0035 at the end of training. Then, we evaluate the performance of the model using the rest 25% samples as the test dataset. To demonstrate the performance of our model, we select several prediction results from the test dataset that display a variety of distribution profiles and compare with their corresponding ground truth in Fig. 4. By comparing the predicted results with the corresponding boundary conditions, we observe that our model captures the distribution feature at boundary (high concentration at top edge in Fig. 4A and low concentration at left edge in Fig. 4B) and gives the prediction following the boundary condition. We also find in Fig. 4C–E that the fixed-size hole is accurately restored in the prediction results. The comparison of time-serial results in each example of Fig. 4 also shows that the dynamic change of distribution can be predicted with good accuracy. Even when the distribution changes dramatically in a short time (*t* = 0.3 to 0.7 in Fig. 4D or *t* = 0.1 to 0.9 in Fig. 4E), the model is still capable of providing convincing prediction with around 1% accuracy decrease.

In addition, we study the influence of the *K*/*D* value, which is the ratio between the reaction and diffusion process, on the concentration distribution results and the training of our model. We first plot the error vs *K*/*D* on test data (see Supplemental Information; Fig. S2) and find the *K*/*D* value of most test samples falls in the range of [0, 500]. Then, we compare several time-serial samples with the same boundary condition to investigate the effect of the *K*/*D* value. Given the same boundary condition as in Fig. 4A, the concentration distribution varies with the *K*/*D* value (see Supplemental Information; Fig. S3). When the *K*/*D* ratio increases, the reaction becomes dominant in the reaction-diffusion system and the time needed to reach steady state drops quickly. The large *K*/*D* value leads to the wide spread of the substance and further decreases the accuracy of our model, as shown in Fig. S3A,B. The hole inside the geometry also brings difficulty to the prediction by making the distribution more complex. To study the influence of hole location on the prediction accuracy, we plot the error vs the location of hole plot on test data (see Supplemental Information; Fig. S4). The plot shows that the test sample has poor performance when the hole locates closer to the domain boundary. We also find that the *K*/*D* values are similar in Fig. S3C,D, but different locations of the hole contribute to two completely different distribution profiles in which the wide-spread distribution shows lower accuracy. Therefore, our model can be further improved to handle the complexity of distribution profiles due to different input simulation parameters and geometries.

We then evaluate the MRE on each sample of the test dataset and the trained model can achieve an average error of 3.04% with the maximum error around 30% (see Supplemental Information; Fig. S5). In addition, the error of most samples are located near the mean value line. By using the defined MRE, we also identify five worst prediction results in the test dataset (see Supplemental Information; Fig. S6). We observe that all the worst predictions (with an average error of 29.94%) contain a hole in the geometry, which indicates that our model tends to have lower accuracy in the system with complex distribution because of the hole.

We also compare the accuracy and computational performance of our algorithm with our FEM solver on a PC with a 2.93 GHz quad core CPU and 16 GB RAM. Since the analytical solution for Equation (5) is unknown, we run our FEM solver in a fine 81 × 81 grid to obtain the baseline solution for accuracy evaluation. To compare the computation time, we run the FEM simulation and CNN prediction to solve the reaction-diffusion equation with the same input configurations for 1,000 time steps. The comparison results of two FEM models and two CNN models are summarized in Table 1. We can observe that the simulation time of the full CNN model (0.155s) is two orders of magnitude faster than the full FEM model (46s) while the error only increases by 1.8%. We then coarsen the input mesh of the full FEM model and obtain a simplified FEM model with similar error as the full CNN model (7.56% vs 7.13%), but the full CNN model is still much faster than the simplified FEM model (16s). We also compare our full CNN model with a simplified CNN model to show the computational behaviour of our model. The simplified CNN model handles the dataset created by FEM simulations on a coarse 11 × 11 grid (see Supplemental Information; Fig. S7). We find that the error increases to 11.37% but the simulation time does not improve much, which suggests that the CNN model is more suitable for large size problems to achieve better performance improvement.

**Table 1:** Comparison between CNN models and FEM models.

Model	Training time	Simulation time	Mean error computed using the baseline solution
Full FEM model in Fig. 2	0	46s	5.74%
Full CNN model in Fig. 3	9.3 hrs	0.155s	7.56%
Simplified FEM model (15 × 15 grid)	0	16s	7.13%
Simplified CNN model in Fig. S7	7.6 hrs	0.112s	11.37%

Note that deep learning is not the only way to predict the solution of PDEs, classical fitting methods can also provide a good approximation. Here, we compare our method with the solution estimated by Fourier series. We randomly pick three sets of time-serial data and each set has the same *D* and *K* values. The approximate solution *u*_*approx*_ of the Fourier series form is given by

4 $${u}_{approx}={a}_{0}+\mathop{\sum }\limits_{m=1}^{M}\mathop{\sum }\limits_{n=1}^{N}({a}_{m}cos(m{\omega }_{x}x)+{b}_{m}sin(m{\omega }_{x}x))({c}_{n}cos(n{\omega }_{y}y)+{d}_{n}sin(n{\omega }_{y}y)),$$

where *M*, *N* are set to be 4 and *a*_0_, *a*_*m*_, *b*_*m*_, *c*_*n*_, *d*_*n*_, *ω*_*x*_, *ω*_*y*_ are to-be-determined parameters. We adopt the least-square method to fit the data at each time step and compare with the prediction results from our model (see Supplemental Information; Fig. S8). We find that deep learning prediction has higher accuracy than the Fourier series approximation. Though Fourier series can estimate the location of the hole (Fig. S8C), it fails to capture the size of the hole (*t* = 0.4 in Fig. S8B) and sometimes it even fails to capture the existence of the hole (Fig. S8A). The possible reason is that the classical fitting method needs a good approximation function to obtain high accuracy and Fourier series may not be a good choice to fit the data. Whereas the structure of neural network can be easily modified to fit for more complex distribution and yield better prediction.

## Discussion

In this paper, we develop a CNN model to study the specific one-component reaction-diffusion system based on the data from the FEM simulation. The network adopts the encoder-decoder architecture with five feature parameters sorted into different input channels of CNN, namely the diffusion coefficient, the reaction rate, time, boundary conditions and the geometry configuration. Given input parameters, our model can provide comparatively accurate and two orders of magnitude faster spatiotemporal distribution prediction compared to the FEM simulation results. The crucial features like the location of the hole and boundary conditions are accurately captured by learning the meaning of different labels from input geometry information. More surprisingly, the CNN model can even predict accurate distribution result at certain time without iterative process, which we believe that the embedded time channel instructs the model to learn the time-dependent behaviour of the reaction-diffusion system. Overall, our well-trained CNN model can provide efficient spatiotemporal distribution prediction of the specific reaction-diffusion system. Compared to FEM simulation, Our deep learning model avoids the expensive solving and iteration processes and generates distribution result directly. When designing a reaction-diffusion process with desired distribution result, the rapid feedback from the design tool is quite important and our model is undoubtedly competent for this task. In addition, our method can also be extended to study more complex reaction-diffusion systems using dataset from experiment observation other than FEM simulation. In this case, more parameters should be introduced in the network architecture to fit for the increasing complexity of the reaction-diffusion system. Suppose the model is trained on the experimental data, the model evaluation may be difficult due to the limited amount of data with ground truth. One common way is to perform cross validation on training data with the limitation on completely ignoring the testing data. Other approaches such as reverse testing framework^39^ and intersection-validation^40^ are suitable in this situation to perform model evaluation utilizing the testing data.

In summary, our model shows multiple novel aspects within both the representation of the data and the architecture of the network used. The introduction of time as a channel for inference, the representation of solid boundaries and geometries into CNN, the conditioning of the diffusion constant or any other material properties and usage of that in combination with other parameters such as time are some of new representation aspects of the project. In addition, using encoder-decoder architecture for time-dependent prediction with multiple channels of the data and their interdependencies is another architectural advance we have performed in this article. Compared to most finite element models or any engineering modeling, we are in essence approximating functions and since neural networks are universal function approximators, all models can be considered as neural networks. However, the convolution operation and the robust feature detection along with the architecture of U-Net which is an encoder-decoder network made it possible to create a surrogate model using neural networks which is faster than conventional FEM models. Our work also has its limitation which we will address in our future work. Based on results from our model, we find that the learning performance is influenced by the *K*/*D* ratio and the geometry configuration. Our model becomes unstable when more features are included, which suggests that we need to increase the complexity of the network to improve the performance. Regarding to the geometry configuration, we only include one hole with fixed rectangle shape but different locations in our dataset and holes with more complex shape should also be included in our model. In addition, we only consider a simple one-fourth arc pipe geometry in 2D domain and we will include more complex geometry to improve the applicability of our model. Despite these limitations, our model directly predicts the dynamic concentration distribution of the reaction-diffusion system and provides a powerful tool for further study in this field.

## Methods

### Problem setting

To specify the problem, we assume a one-component reaction-diffusion system inside a one-fourth arc pipe in 2*D* domain (Fig. 2A). This geometry is selected to derive asymmetric concentration distributions and introduce the learning of geometry features to the model. The arc pipe has the inner radius *R*_*i*_ = 5 and the outer radius *R*_*o*_ = 10. The concentration change of one chemical substance in space and time can be described using the following reaction-diffusion equation,

5 $$\frac{\partial u}{\partial t}=D\Delta u+R(u),$$

where *D* is the diffusion coefficient, *u* = *u*(*x*, *y*, *t*) is the concentration of the substance, *R*(*u*) is the reaction term. Here we select *R*(*u*) = *Ku*^2^(1 − *u*), where *K* is the reaction rate and this equation is also referred to as the Zeldovich equation^41^ used in the combustion theory.

The initial condition is set to be *u*(*x*, *y*, 0) = 0. We apply the Dirichlet boundary condition at four edges of the pipe (Fig. 2A),

6 $$u(x,y,t)=\left\{\begin{array}{ll}{u}_{1}, & x=0,\\ {u}_{2}, & r={R}_{i},\\ {u}_{3}, & y=0,\\ {u}_{4}, & r={R}_{o},\end{array}\right.$$

where $r=\sqrt{{x}^{2}+{y}^{2}}$ is the distance between the point (*x*, *y*) to the center of the arc pipe.

To consider the effect of different geometry configurations, we introduce a hole with fixed size at different locations of the pipe (black dash box in Fig. 2A). At the boundary of the hole, we also apply the Dirichlet boundary condition,

7 $${u}_{hole}={u}_{5},$$

and we set *u*_5_ = 0 to simplify the problem.

### Data generation

The dataset of reaction-diffusion system can be collected from experiments or computer simulation results. For fast and flexible dataset generation, we develop a FEM-based solver to solve Equation (5) and obtain dynamic concentration results for the system. We create a 21 × 21 quadrilateral mesh (Fig. 2B) for the one-fourth arc pipe as the input of the FEM solver and one sample result is shown in Fig. 2C. Four types of simulation parameters are considered in our model, namely the diffusion coefficient *D*, the reaction rate *K*, time *t* and boundary values *u*_1_~*u*_4_. To include the effect of geometry on the concentration result, we put a hole consisting of 2 × 2 elements to 20 different locations inside the pipe and obtain 21 different geometries including one geometry without hole. For each geometry, we generate 500 different sets of *D*, *K* and *u*_1_~*u*_4_ and solve the equation for 1,000 time steps (time step size *δt* = 0.01 *s*). *D* and *K* are randomly selected in range [0, 10] and *u*_1_~*u*_4_ are randomly selected in range [0, 1]. The result of every 10 steps is extracted and we finally create the dataset containing 1,050,000 samples. Then we perform data processing to fit the dataset into our neural network with tensor data format.

For each FEM simulation result, we directly extract the nodal concentration value from the mesh as the input data instead of using the concentration color map. Since the single pipe geometry is topologically equivalent to a rectangle, we map the concentration distribution result from the physical domain (Fig. 2A) to a unit square in the parametric domain (Fig. 2D). In this case, the topological mapping between physical space coordinates (*x*, *y*) and parametric space coordinates (*ξ*, *η*) is defined as

$$\left\{\begin{array}{l}\xi =(r-{R}_{i})/({R}_{o}-{R}_{i}),\\ \eta =2\theta /\pi ,\end{array}\right.$$

where $r=\sqrt{{x}^{2}+{y}^{2}}$ is the distance between the point (*x*, *y*) to the center of the arc pipe and $\theta =\arctan (y/x)$ is the angle between vector (*x*, *y*) and *x*-axis.

The nodal concentration values are then stored in a 21 × 21 matrix as one channel of the data. In particular, the nodal concentration values are set to 0 inside the hole region. Regarding to the input boundary and initial condition information, we use a matrix with the same size and store the boundary values in it with the hole region setting to be −2 and the region exterior to the hole setting to be −1, shown in Fig. 2E. For the other three feature parameters *D*, *K* and *t* (scalars), we create three 21 × 21 matrices to store each of them. The matrix representation of the feature parameters can be easily fitted in the convolutional operation during the encoding process. The usage of matrix representation also accounts for the possible spatial variant feature parameters. In particular, if *D* and *K* are invariant scalars, they can also be stored into two 2 × 2 matrices and concatenated with the hidden layer obtained at the end of the encoding process (Fig. 3).

In summary, we generate four input matrices including the feature information (*i.e*. different geometries and parameter configurations) and one output matrix including the dynamic concentration result.

## Supplementary information

Supplementary Information.

## Data Availability

The source code for our neural network is available for download from a public software repository located at https://github.com/truthlive/RDCNN. All data generated during this study can be reconstructed by running the source code.
