# Supplementary material for: Reaction diffusion system prediction based on convolutional neural network
Source: Sci Rep. 2020 Mar 3;10:3894. doi: 10.1038/s41598-020-60853-2 (PMC7054402; doi:10.1038/s41598-020-60853-2)
Supplement: Supplementary file 1 — Supplementary Information. [file 41598_2020_60853_MOESM1_ESM.pdf]

Supplementary Information
Reaction diffusion system prediction based on convolutional neural network

Angran Li, Ruijia Chen, Amir Barati Farimani and Yongjie Jessica Zhang

Supplementary Figures

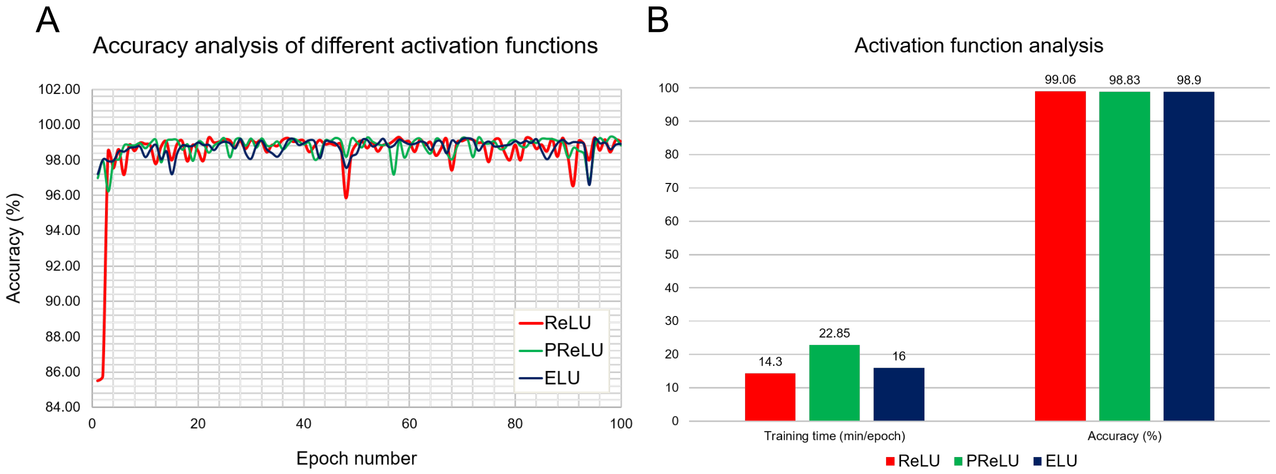

Figure S1. The experiment result of using different activation functions (ReLU, PReLU and ELU) in the proposed network. (A) The epoch-accuracy curve for different activation functions. (B) The comparison of test accuracy and training time for different activation functions.

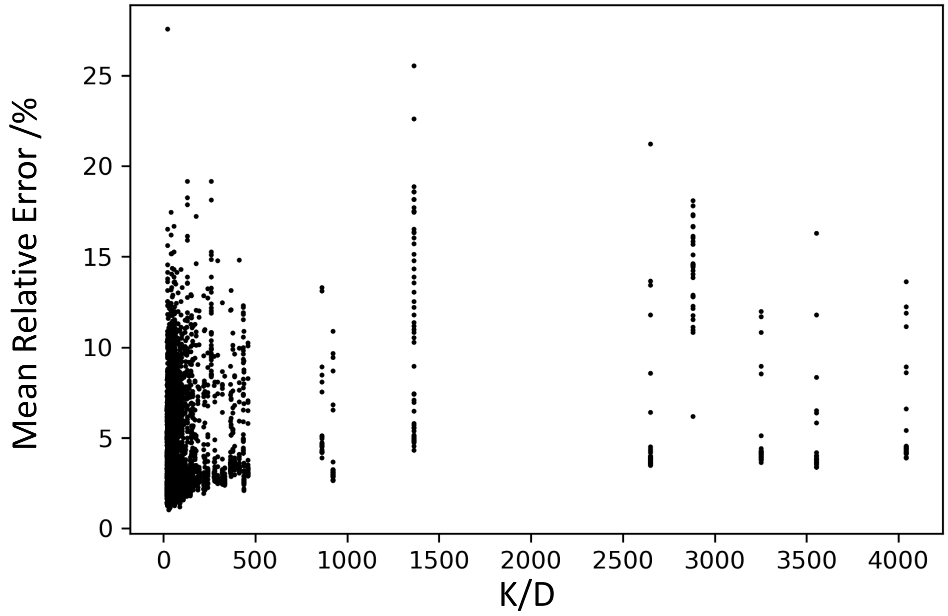

Figure S2. tatistical accuracy vs the K/D value evaluated on the test dataset. Each data point represents one sample. The K/D value of most test samples falls in the range of $[0, 500]$.

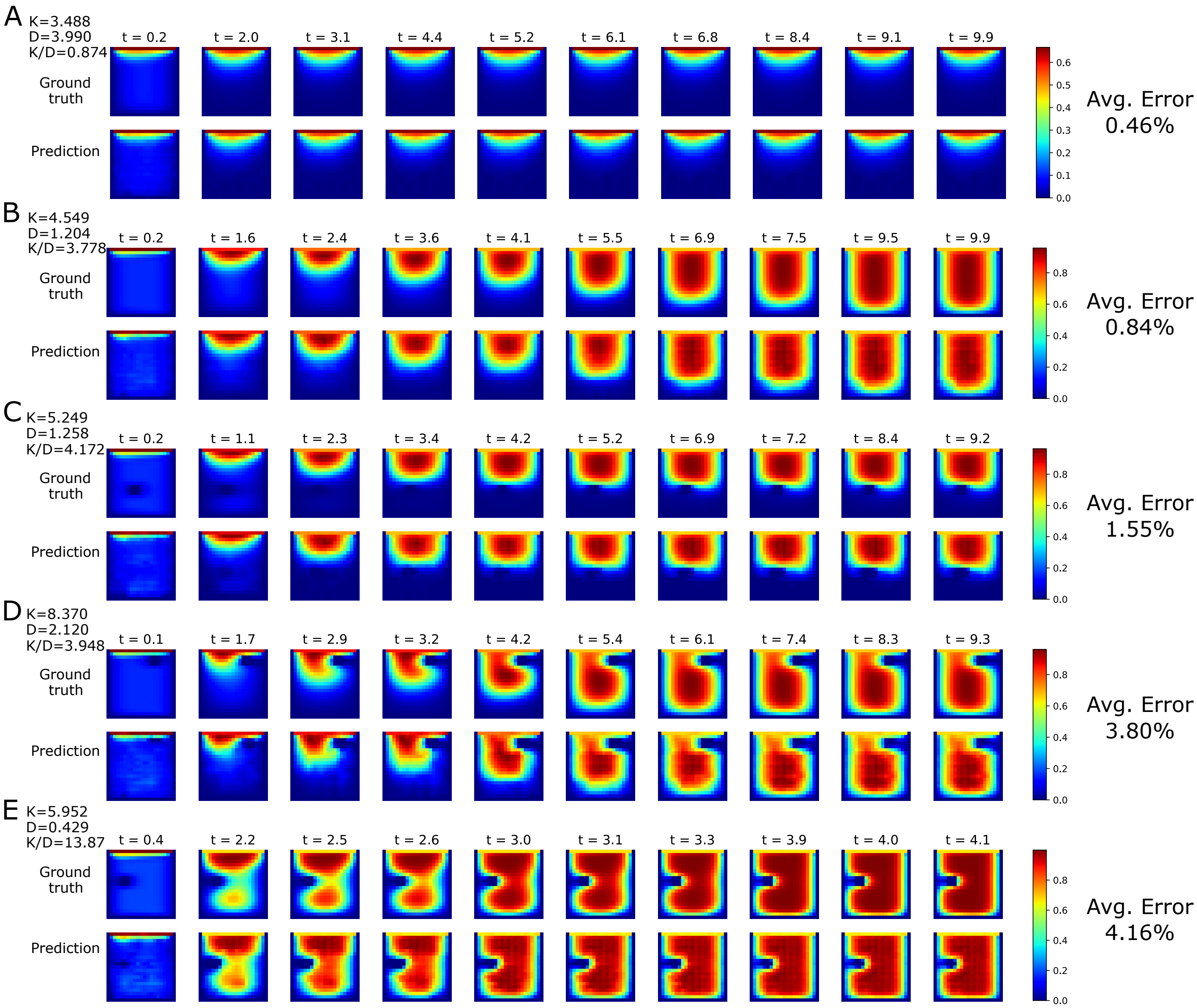

Figure S3. The concentration distribution results of five different parameter and geometry configurations are compared with the same boundary condition ($u_1 = 2/3, u_2 = u_3 = u_4 = 0$). (A, B) The same geometry without hole; and (C-E) different geometries with the hole at different locations. For each configuration, the ground truth results and predicted results are shown in the top row and the bottom row, respectively. The average error of each result is compared to illustrate that high K/D value and the existence of the hole decreases the accuracy of the prediction.

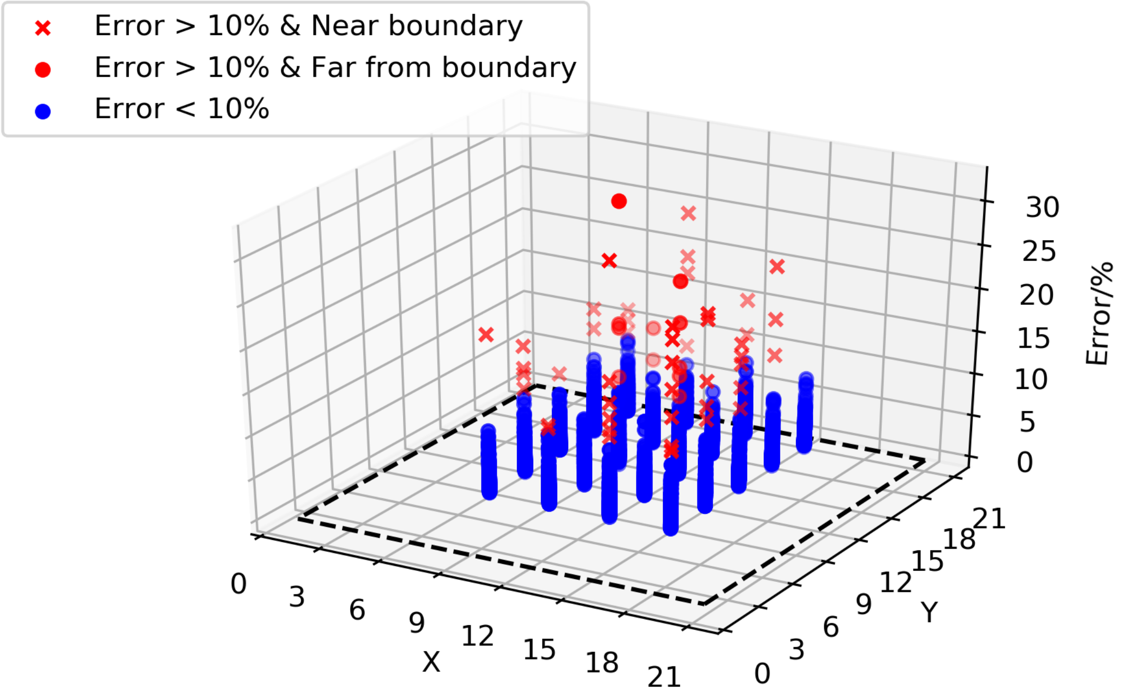

Figure S4. Statistical accuracy vs location of the hole evaluated on the test dataset. The black dash line represents the domain boundary. Each data point represents one sample. The samples with error over and below 10% are shown in red and blue, respectively. Among all the red data points, the cross data points represent samples containing a hole near the domain boundary. The model shows poor performance when the hole locates closer to the domain boundary.

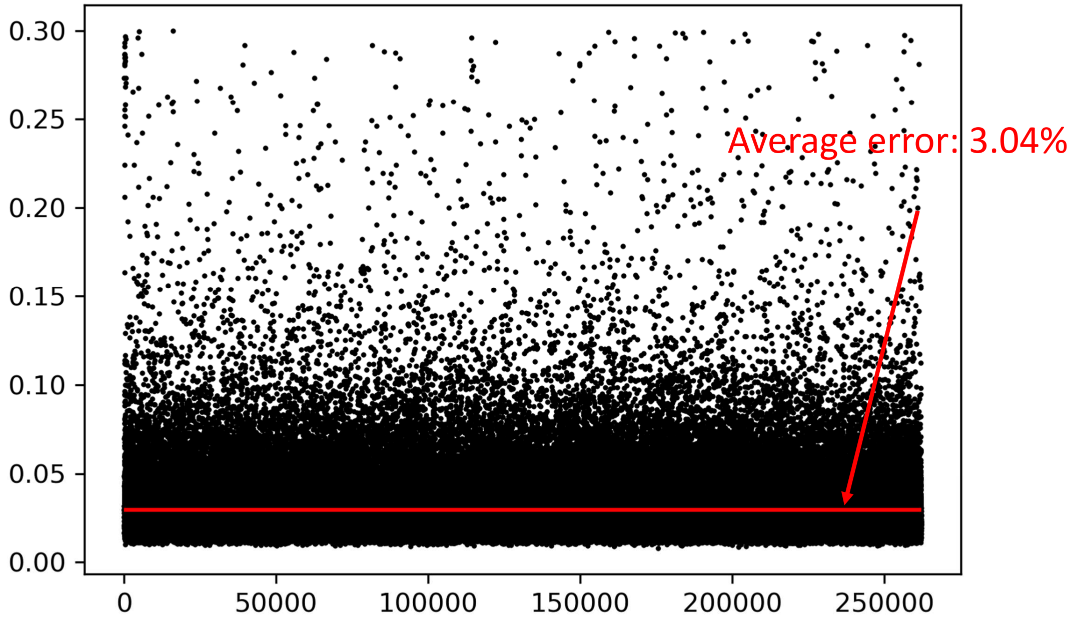

Figure S5. Statistical accuracy of CNN model evaluated on the test dataset.

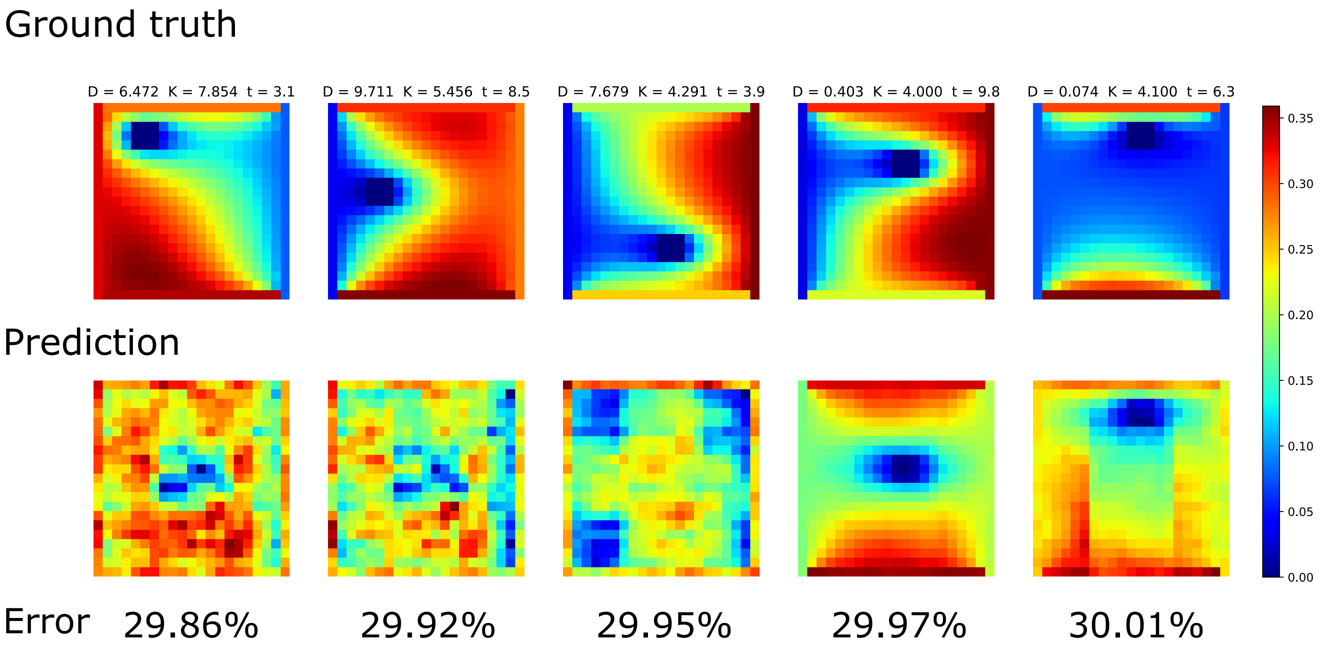

Figure S6. Five worst prediction results on the test dataset. The ground truth results and the predicted results are shown in the top row and the bottom row, respectively.

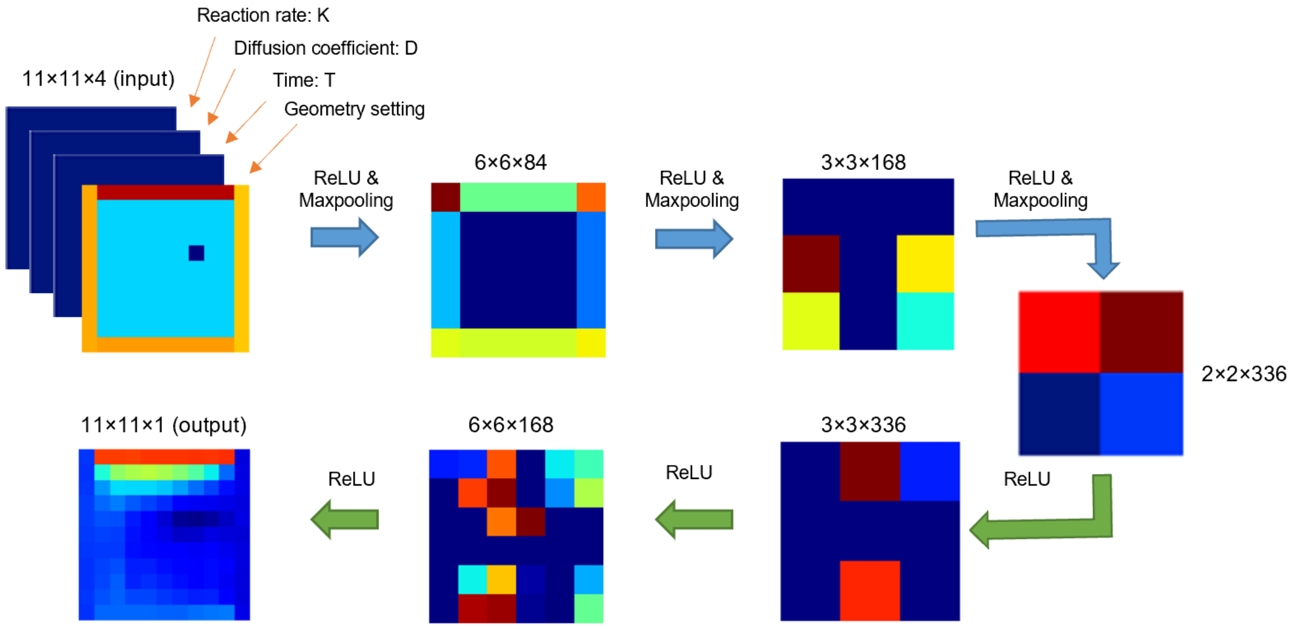

Figure S7. The simplified CNN with encoder-decoder architecture. Blue and green arrows represent encoding and decoding, respectively. A representative output for each layer is shown for both encoding and decoding layers.

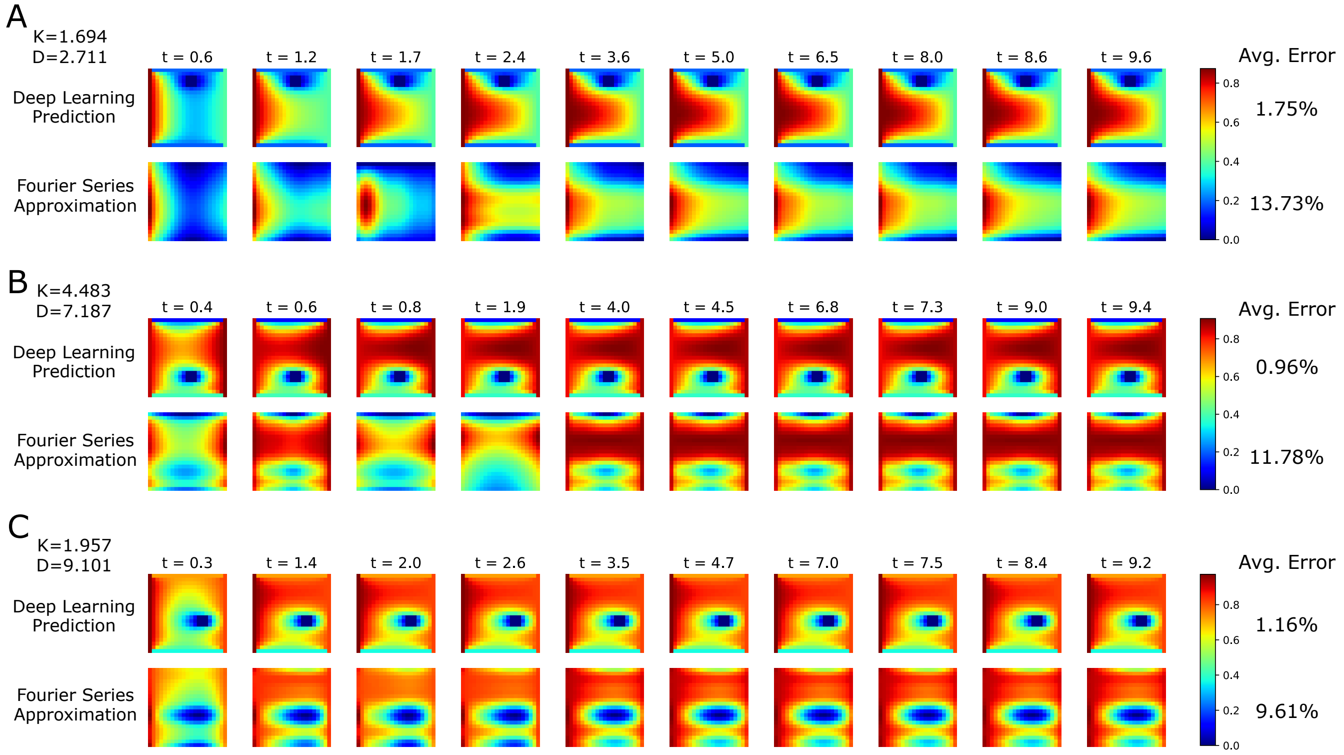

Figure S8. The comparison between deep learning prediction and Fourier series approximation. In each subfigure, the time-serial results with the same K and D values are compared. The deep learning results and the Fourier series approximation results are shown in the top row and the bottom row, respectively. The average error of each set of results is listed at the end of each row. In all cases, deep learning prediction shows higher accuracy. (A, B) Fourier series approximation fails to capture the hole; and (C) Fourier series approximation captures the hole with inaccurate hole size.
